# Neoadjuvant chemotherapy versus neoadjuvant chemoradiotherapy for locally advanced oesophageal squamous cell carcinoma: a single-Centre, open-label, randomized, controlled, clinical trial (HCHTOG1903)

**DOI:** 10.1186/s12885-020-06824-2

**Published:** 2020-04-15

**Authors:** Hai-Bo Sun, Wen-Qun Xing, Xian-Ben Liu, Yan Zheng, Shu-Jun Yang, Zong-Fei Wang, Shi-Lei Liu, Yu-Feng Ba, Rui-Xiang Zhang, Bao-Xing Liu, Cheng-Cheng Fan, Pei-Nan Chen, Guang-Hui Liang, Yong-Kui Yu, Qi Liu, Hao-Ran Wang, Hao-Miao Li, Zhen-Xuan Li, written on Henan Cancer Hospital Thoracic Oncology Group (HCHTOG)

**Affiliations:** 1 Department of Thoracic Surgery, The Affiliated Cancer Hospital of Zhengzhou University (Henan Cancer Hospital), No. 127 Dongming Road, Zhengzhou, 450008 China; 2 Department of Medical Oncology, The Affiliated Cancer Hospital of Zhengzhou University, Henan Cancer Hospital (Henan Cancer Hospital), Zhengzhou, China; 3 Department of Radiation Oncology, The Affiliated Cancer Hospital of Zhengzhou University, Henan Cancer Hospital (Henan Cancer Hospital), Zhengzhou, China

**Keywords:** Oesophagus, Squamous cell carcinoma, Neoadjuvant chemotherapy, Neoadjuvant chemoradiotherapy

## Abstract

**Background:**

Neoadjuvant therapy plus oesophagectomy has been accepted as the standard treatment for patients with potentially curable locally advanced oesophageal cancer. No completed randomized controlled trial (RCT) has directly compared neoadjuvant chemotherapy and neoadjuvant chemoradiation in patients with oesophageal squamous cell carcinoma (ESCC). The aim of the current RCT is to investigate the impact of neoadjuvant chemotherapy plus surgery and neoadjuvant chemoradiotherapy plus surgery on overall survival for patients with resectable locally advanced ESCC.

**Methods:**

This open label, single-centre, phase III RCT randomized patients (cT2-T4aN + M0 and cT3-4aN0M0) in a 1:1 fashion to receive either the CROSS regimen (paclitaxel 50 mg/m^2^; carboplatin (area under the curve = 2), q1w, 5 cycles; and concurrent radiotherapy, 41.4 Gy/23 F, over 5 weeks) or neoadjuvant chemotherapy (paclitaxel 175 mg/m^2^; and cisplatin 75 mg/m^2^, q21d, 2 cycles). Assuming a 12% 5-year overall survival difference in favour of the CROSS regimen, 80% power with a two-sided alpha level of 0.05 and a 5% dropout each year for an estimated 3 years enrolment, the power calculation requires 456 patients to be recruited (228 in each group). The primary endpoint is 5-year overall survival, with a minimum 5-year follow-up. The secondary endpoints include 5-year disease-free survival, toxicity, pathological complete response rate, postoperative complications, postoperative mortality and quality of life. A biobank of pre-treatment and resected tumour tissue will be built for translational research in the future.

**Discussion:**

This RCT directly compares a neoadjuvant chemotherapy regimen with a standard CROSS regimen in terms of overall survival for patients with locally advanced ESCC. The results of this RCT will provide an answer for the controversy regarding the survival benefits between the two treatment strategies.

**Trial registration:**

NCT04138212, date of registration: October 24, 2019.

## Background

Oesophageal cancer is a prevalent malignancy worldwide and causes more than 400,000 annual deaths worldwide [1]. Oesophagectomy still plays an important role in the treatment of oesophageal cancer. However, surgery alone is often accompanied by high recurrence and metastasis rates in patients with locally advanced oesophageal cancer, and this has brought about a shift in the management strategy from locoregional therapy alone to multimodality regimens [2]. To decrease locoregional and distant recurrences and improve survival, neoadjuvant therapies have been tested [3, 4]. In large parts of the Western world, neoadjuvant chemoradiotherapy (nCRT) plus surgery has been adopted as a standard treatment for patients with locally advanced oesophageal cancer based on the CROSS study [5]. However, some countries in Asia, especially Japan, advocate the use of neoadjuvant chemotherapy (nCT) as a standard treatment based on the JCOG9907 study [6].

Studies directly comparing nCT with nCRT are lacking. The Preoperative Chemotherapy or Radiochemotherapy in Esophagogastric Adenocarcinoma Trial (POET), in which nCT (cisplatin, 5-FU and leucovorin) and nCRT (cisplatin and etoposide with concurrent radiation therapy 30 Gy/15 Fr) were compared in patients with adenocarcinoma of the oesophagogastric junction, has been the only phase III RCT to address this question [7]. It was initially planned that a total of 354 patients would be enrolled; however, the study was prematurely closed due to low accrual. Finally, a total of 126 patients were randomly assigned, and 119 eligible patients were evaluated. The pathological complete response rate (pCR) was significantly improved (15.6% vs 2%, *P* = 0.03) with the addition of radiation. The study failed to show any significant difference in the endpoint (overall survival, OS), although the 3-year survival rate was significantly prolonged in the nCRT group compared to the nCT group, indicating the possible usefulness of preoperative chemoradiotherapy.

As there are no results from a head-to-head RCT, controversy still exists regarding which therapy is superior in patients with oesophageal squamous cell carcinoma (ESCC). To compare the outcomes between nCRT and nCT in patients with locally advanced ESCC, we initiated this RCT.

## Methods and design

### Study design (Fig. 1)

**Fig. 1:**
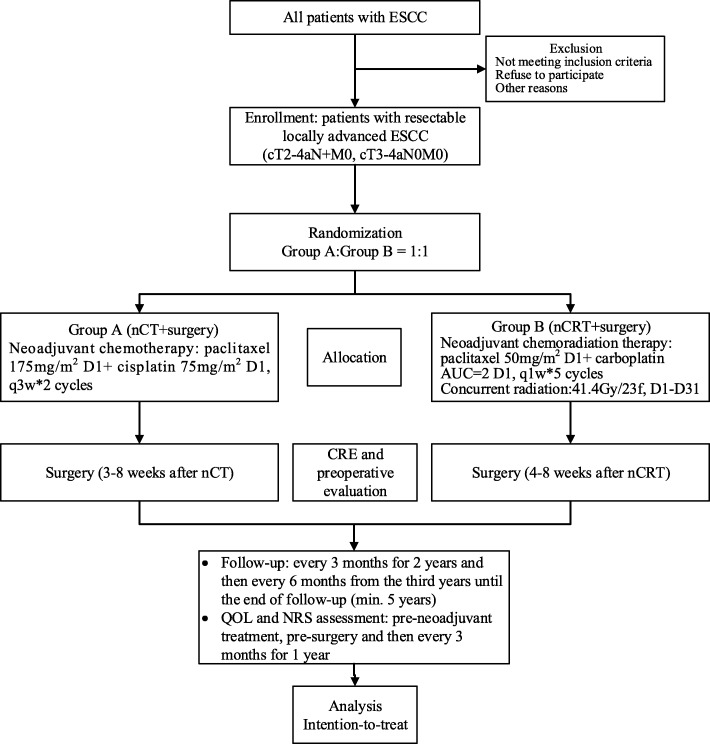
Flowchart of the study. Abbreviations: AUC = area under the curve; CRE = clinical response evaluation; ESCC = oesophageal squamous cell carcinoma; nCRT = neoadjuvant chemoradiotherapy; nCT = neoadjuvant chemotherapy; QOL = quality of life

HCHTOG1903 is a single-centre phase III two-arm open-labelled RCT. Eligible patients are randomly assigned to nCT or nCRT (CROSS protocol) and surgery with a 1:1 allocation ratio (Fig. 1). The purpose of this study is to confirm the superiority of a standard nCRT regimen in terms of OS over nCT as preoperative therapy for resectable locally advanced ESCC.

### Primary endpoint

The primary endpoint was OS in all randomized patients. OS is defined as the number of months from randomization to death from any cause, and patients are censored on the last day they are known to be alive.

### Secondary endpoints

1. Disease-free survival (DFS) time: The time from the date of randomization to the date of first recurrence (local, regional or distant) or death.
2. pCR rate: The degree of tumour regression is classified into four categories according to the modified Ryan scheme: grade 0, no viable cancer cells, including lymph nodes; grade 1, single cells or rare small groups of cancer cells; grade 2, residual cancer with evident tumour regression but more than single cells or rare small groups of cancer cells; and grade 3, extensive residual cancer with no evident tumour regression [8].
3. Postoperative complications: The definition of each complication is listed in supplemental file 1. All postoperative complications will be captured for up to 90 days after surgery and graded according to the Clavien–Dindo classification [9].
4. Postoperative mortality: 30-day and 90-day postoperative mortality.
5. Adverse events (AE): Chemoradiation/chemotherapy-related adverse events are collected according to the National Cancer Institute’s Common Terminology Criteria for Adverse Events, version 5.0. All non-serious and serious AEs occurring in each patient will be reported up to 3 weeks after the last dose of chemotherapy or radiation therapy has been received.
6. Quality of life (QOL) assessment and nutritional risk screening (NRS): The European Organization for Research and Treatment of Cancer (EORTC) questionnaires C30 and OES18 were used to assess QOL. The nutritional risk score is calculated according to the data collected on the NRS 2002 form. QOL and NRS were assessed at randomization, in the middle of neoadjuvant therapy, 1 week before surgery and 2 weeks, 1 month, 3 months, 6 months, 9 months, and 12 months after surgery.

### Patient selection

Patients in the Affiliated Cancer Hospital of Zhengzhou University (Henan Cancer Hospital) with histologically proven resectable thoracic ESCC after preoperative staging will be considered for enrolment in the trial. All patients undergo pre-treatment staging according to the 8th UICC TNM system [10]. This included a history taking; physical examination; pulmonary-function tests; electrocardiogram (ECG); routine haematologic and biochemical tests; endoscopic ultrasonography with biopsies; upper gastrointestinal contrast; cardiac and cervical ultrasonography; computed tomography (CT) scans of the brain, thorax, and abdomen; and bone scintigraphy. Positron emission tomography (PET) with fluorodeoxyglucose is used when distant metastasis is suspected. All oesophageal cancer patients will be discussed by a multidisciplinary team (MDT) before any treatment. Written informed consent is obtained from all patients by the doctors in charge prior to participation in the trial.

### Inclusion criteria

Eligible patients must meet all of the following criteria:

1. Histologically proven squamous cell carcinoma.
2. Tumours are located in the thoracic oesophagus.
3. Age is between 18 years and 70 years.
4. ECOG performance status of 0 or 1.
5. Clinical stages cT2-T4aN + M0 and cT3-4aN0M0 based on the 8th UICC TNM system (10).
6. No metastatic cervical lymph nodes.
7. R0 resection is expected by the McKeown minimally invasive oesophagectomy (MIE), open right thoracotomy oesophagectomy or hybrid approaches after MDT discussion.
8. No prior chemotherapy, radiotherapy or hormonal therapy was administered against any cancers.
9. Adequate cardiac function: ejection fraction ≥50%.
10. Adequate respiratory function: FEV1% ≥ 50% and DLCO ≥50%.
11. Adequate bone marrow function: white blood cell count ≥4 × 10^9^/L; absolute neutrophil count (ANC) ≥ 1.5 × 10^9^/l; platelets ≥100 × 10^9^/L; haemoglobin ≥9 g/dl.
12. Adequate liver function: serum bilirubin ≤1.5 × upper limit of normal (ULN); aspartate transaminase (AST) and alanine transaminase (ALT) ≤ 2.0 × ULN (ULN as per institutional standard).
13. Adequate renal function: glomerular filtration rate ≥ 60 ml/min calculated using the Cockcroft-Gault formula.
14. Written consent is obtained.

### Exclusion criteria

Patients meeting any of the following criteria are not eligible for this trial:

1. Synchronous or metachronous (within 5 years) double cancers.
2. Active infection requiring systemic therapy.
3. Tumour width > 5 cm.
4. Patients requiring systemic steroid medication.
5. Patients with contraindications for oesophagectomy.
6. Psychiatric disease.
7. Patients in whom gastric tubes cannot be used for reconstruction after oesophagectomy.
8. Pregnant or lactating women or women of childbearing potential.
9. Hypersensitivity for paclitaxel, cisplatin or carboplatin drugs.

### Randomization

A clinical research coordinator is responsible for randomization. Computerized randomization lists are created, and the results are placed in sealed opaque envelopes. After confirmation of the eligibility criteria, the patients are randomly allocated (1:1) to the nCT group or nCRT group. The intervention in this study is not blinded.

### Treatment regimens (Fig. 2)

#### Arm A - neoadjuvant chemotherapy

**Fig. 2:**
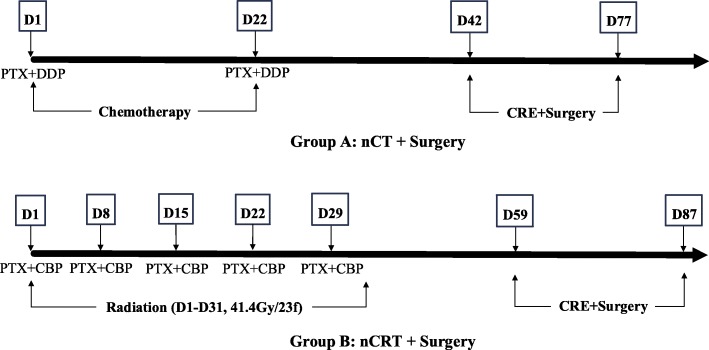
Treatment plans for the two groups. Abbreviations: CBP = carboplatin; CRE = clinical response evaluation; DDP = cisplatin; nCRT = neoadjuvant chemoradiotherapy; nCT = neoadjuvant chemotherapy; PTX = paclitaxel

Patients in arm A receive 2 cycles of chemotherapy prior to surgery. The detailed regimen runs are as follows: paclitaxel 175 mg per square metre of body-surface area on day 1 and cisplatin 75 mg per square metre of body-surface area on day 1 (cisplatin can be divided into 3 days). Chemotherapy was repeated every 3 weeks (Fig. 2). The doses of paclitaxel and cisplatin will be reduced to 75% of the planned dose if any grade 4 toxicity appears during chemotherapy.

#### Arm B - neoadjuvant chemoradiotherapy

Patients in arm B receive chemotherapy and concurrent radiotherapy prior to surgery. On days 1, 8, 15, 22, and 29, the patient will receive 5 cycles of chemotherapy, carboplatin (area under the curve (AUC) = 2 (calculated using the Calvert formula)) and paclitaxel at a dose of 50 mg per square metre of body-surface area are administered intravenously (Fig. 2). If the white blood cell (WBC) count is < 1.0 × 10^9^/L and/or platelets are < 50 × 10^9^/L, chemotherapy is delayed by 1 week until recovery to above these values. The doses of paclitaxel and carboplatin will be reduced to 75% of the planned dose if any grade 4 toxicity appears during chemoradiotherapy. Concurrent radiotherapy with 41.4 Gy is given in 23 fractions of 1.8 Gy each, with 5 fractions administered per week, starting on the first day of the first chemotherapy cycle. The gross tumour volume is defined as the volume of the primary tumour and the regional metastatic lymph nodes. The clinical target volume (CTV) includes the primary tumour with a 3-cm cranio-caudal margin, metastatic lymph nodes and regional lymph nodes. The planning target volume is defined as the CTV plus a 0.5–0.8 cm isotropic margin. All patients are treated by means of external-beam radiation using the 3-D conformal radiation technique.

### Surgery

After neoadjuvant therapy, patients will receive the same examinations as pre-treatment. Endoscopy evaluation is not necessary after neoadjuvant therapy. In the nCT group, the operation will be performed between 3 and 8 weeks following completion of nCT, and in the nCRT group, the operation will be performed between 4 and 8 weeks following completion of nCRT (Fig. 2). MIE, open right thoracotomy oesophagectomy or hybrid approaches with a total 2-field lymphadenectomy will be performed. A gastric tube will be used to reconstruct the digestive tract after oesophagectomy. Oesophagectomy with a transhiatal or left thoracotomy approach is not acceptable because of the limited capacity for lymph node dissection, especially for the lymph nodes along the bilateral recurrent laryngeal nerve, with the two approaches. All postoperative complications will be recorded on the case report form for up to 90 days after surgery.

### Follow-up

All randomized patients will be followed up for at least 5 years after patient accrual is completed. The first follow-up visit will occur 1 month after surgery. From then on, follow-up visits will occur at 3 months, 6 months, 9 months and 12 months for the first year; QOL and NRS will also be evaluated. In the second year, follow-up visits will occur every 3 months and every 6 months from the third year until the end of follow-up. The detailed examination items include a CT scan of the thorax and upper abdomen, and ultrasonography of the neck. PET/CT is used when distant metastasis is suspected. Recurrence of disease should be documented by appropriate imaging and biopsies where appropriate.

### Translational research

The clinical trial includes tissue and blood sample collection for translational research. Trial participants will be asked to provide additional optional written consent for sample collection. The standard tissue sample consists of a pre-treatment biopsy tumour tissue and normal mucosa and a postoperative biopsy of tumour tissue and normal mucosa. Fasting blood samples are obtained at the time of the pre-treatment evaluations, in the middle of neoadjuvant therapy, pre-surgery, and 1 week after surgery. The blood samples are centrifuged at 1300×g for 10 min to remove cells and debris. All samples will be stored at the Henan Cancer Hospital Tissue Bank at − 80 °C for future translational research.

### Statistical analysis

We assumed 5-year OS with preoperative chemotherapy to be 30% and expected a 12% increase in 5-year OS with preoperative chemoradiotherapy. We set a two-sided type I error of 5%, a power of 80% and a 5% drop out each year for an estimated 3 years of enrolment. A total of 456 patients (228 patients in each group) will be enrolled in this study according to the estimate calculated with PASS 11 statistical software (NCSS, LLC. Kaysville, Utah, USA).

An interim analysis is planned after recruitment of approximately 230 patients. Data will be analysed according to the intention-to-treat (ITT) principle with all randomized patients. A per-protocol analysis, excluding patients who did not sufficiently comply with the protocol, will supplement the ITT analysis as a secondary analysis. Comparisons between the groups will be made with the chi-square test and Fisher exact test for categorical parameters, while with Student’s t tests or Mann–Whitney U tests will be used for comparisons of continuous variables. Survival rates in the two treatment arms will be estimated by the Kaplan-Meier method. Then, the Cox proportional hazard model and the log rank test will be used to evaluate the independent survival factors. All tests will be two-sided. The significance level is set at 0.05.

### Funding, registration, ethical considerations and current status

This study was funded by the Province-Ministry Co-construction Project of Health Committee of Henan Province (SB201901108). The study protocol (version 2.0) has also undergone peer-review by this government funding body. The protocol (version 2.0) was reviewed and approved by the Affiliated Cancer Hospital of Zhengzhou University (Henan Cancer Hospital) Ethics Committee (No. 2019082223) in September 30, 2019. The study will be conducted in accordance with ethical principles founded in the Declaration of Helsinki [11]. Data are collected using the individual trial case number on case report forms and personal information will not be individually identifiable. The Department of Clinical Trial Management of the Affiliated Cancer Hospital of Zhengzhou University (Henan Cancer Hospital) will be responsible for reviewing the trial data approximately every 6 months. The corresponding author (Dr. WQX) will be responsible for design and conduct of HCHTOG1903.The final trial dataset will be available to principle investigators.

This study was registered before the start of recruiting at ClinicalTrials.gov in October, 2019 (registration number: NCT 04138212). Our study began to recruit in October, 2019 and it is still at the stage of recruiting.

## Discussion

To date, neoadjuvant therapy (chemotherapy or chemoradiotherapy) plus oesophagectomy have been adopted as standard treatment strategies for patients with potentially curable locally advanced oesophageal cancer. Although most studies favour nCRT, some prefer nCT without radiation. To date, evidence is insufficient to determine whether combined nCT plus surgery or nCRT plus surgery is the most beneficial treatment strategy for patients with ESCC. Therefore, a head-to-head comparison between preoperative chemotherapy and preoperative chemoradiotherapy is being pursued. To compare the survival benefits between nCT and nCRT in patients with locally advanced ESCC, we designed this RCT.

Chemotherapy acts both locally and systemically by downstaging the primary tumour to increase the chance of radical resection and elimination of micrometastases and decrease the risk of developing distant metastases. Over the last three decades, several randomized trials have compared nCT followed by surgery with surgery alone. The largest trial including mostly oesophageal cancer patients undergoing nCT (cisplatin and fluorouracil) followed by surgery (*n* = 400) versus surgery alone (*n* = 402) was the British OEO2 trial [12, 13]. The OEO2 study showed a survival benefit with nCT, with R0 resection rates and 5-year OS significantly improved from 17.1 to 23.0% (*P* = 0.03) [12]. These treatment results were consistent in both adenocarcinoma and squamous cell carcinoma patients. However, the results of the OEO2 trial were not confirmed by the US Intergroup trial 113 [14, 15]. The US study randomized 213 oesophageal cancer patients to perioperative chemotherapy (cisplatin + fluorouracil) and 227 patients to surgery alone. Patients undergoing preoperative chemotherapy followed by surgery or surgery alone had similar R0 resection rates (59% vs 63%) and 5-year OS (22% vs 19%) [14]. The MAGIC trial was published in 2006, and the results showed that after the addition of perioperative chemotherapy consisting of epirubicin, cisplatin and fluorouracil, the 5-year OS significantly improved from 23 to 36% (*P* = 0.009). However, since only patients with adenocarcinoma were enrolled and the majority of the patients (75%) had gastric cancer, the results of this study cannot be extrapolated to patients with oesophageal cancer, especially for patients with ESCC [16]. The Japanese Clinical Oncology Group (JCOG) conducted a trial JCOG9907 to ascertain the optimal timing of perioperative chemotherapy [6]. A total of 330 patients with ESCC were randomized either to postoperative or preoperative chemotherapy with cisplatin and fluorouracil. In an interim analysis, the Data and Safety Monitoring Board recommended early publication of the results after the OS was shown to be superior in patients undergoing nCT (HR 0.64; 95% CI, 0.45–0.91; *P* = 0.01). The final analyses showed that the 5-year survival was better in the preoperative arm (55% vs 43%) without any additional adverse events [6]. Therefore, in Japan, nCT plus surgery is a standard treatment for locally advanced oesophageal cancer based on the JCOG9907 trial.

nCRT has the advantage of combining chemotherapy and radiation prior to surgery, addressing both locoregional disease and micrometastases. In 1996, the first adequately powered RCT to study the outcomes of nCRT followed by surgery versus surgery alone in patients with oesophageal adenocarcinoma was reported [17]. Between 1990 and 1995, 113 patients were randomized into either nCRT consisting of two cycles of fluorouracil and cisplatin concurrently with 40 Gy radiotherapy in 15 fractions followed by surgery or surgery alone. After the addition of nCRT, the three-year OS significantly improved from 6 to 32% (*P* = 0.01). The Trans-Tasman Radiation Oncology Group (TROG) and the Australasian Gastro-Intestinal Trials Group (AGITG) randomized 256 patients equally to surgery alone or to nCRT followed by surgery. One cycle of cisplatin and fluorouracil was given along with 35 Gy radiation (in 15 days) in the nCRT group. The results of this trial showed no benefit with nCRT in either PFS or OS, although a subset analysis showed superior survival in patients with squamous cell carcinoma. The drawbacks of this study included the suboptimal dose of radiation (35 Gy) and the single cycle of chemotherapy [18]. The CROSS trial randomized 366 patients into the nCRT group (weekly carboplatin and paclitaxel for 5 weeks with a radiation dose of 41.4 Gy in 23 fractions) and a surgery alone group. After nCRT, 92% of patients had R0 resection, compared to 69% in the surgery alone group. This trial demonstrated that 5-year OS improved from 33 to 47% in the nCRT group, and there was a conspicuous prognostic add-on effect of preoperative chemoradiotherapy, particularly for patients with squamous cell carcinoma [19]. The role of nCRT has now been widely accepted as a standard treatment for locally advanced oesophageal cancer in the Western world after the publication of the CROSS trial.

Although many studies have been initiated in the field of neoadjuvant therapy for patients with oesophageal cancer, the controversy regarding the optimal neoadjuvant treatment regimen remains unresolved. There are very few trials directly comparing nCT with nCRT therapy, and some studies involving direct comparisons were of moderate to poor quality [20, 21]. At present, there are some ongoing phase III RCTs comparing nCRT to nCT in patients with locally advanced oesophageal cancer. The ESOPEC trial [22] is a multicentre phase III German study comparing the efficacy of nCRT (CROSS protocol) versus perioperative chemotherapy in oesophageal adenocarcinoma; the endpoints include survival, treatment-related morbidity and quality of life. The Irish Neo-AEGIS trial [23], which is similar to the ESOPEC trial, compares the modified MAGIC protocol with the CROSS protocol in adenocarcinoma of the oesophagus and gastroesophageal junction. At present, there are only two ongoing RCTs exclusively focusing on patients with ESCC: the Chinese CMISG1701 study and the Japanese JCOG1109 study. The CMISG1701 study [24] is a multicentre RCT investigating the safety and efficacy of nCRT plus MIE compared with nCT plus MIE in patients with ESCC. The nCT arm consists of two cycles of preoperative chemotherapy (paclitaxel 135 mg/m2 D1 and cisplatin 75 mg/m2 D1, q3w) before surgery. The nCRT arm consists of a combination of preoperative radiotherapy (40 Gy/20f) and chemotherapy (paclitaxel 50 mg/m2 D1 and cisplatin 25 mg/m2 D1, q1w × 4). A total of 264 patients will be enrolled in this study. Compared with the CMISG1701 trial, our study has a larger enrolment number with a much greater statistical power, and the nCRT regimen used in the CMISG1701 trial is not a widely accepted CROSS regimen. Furthermore, in the CMISG1701 trial, the dose of paclitaxel is relatively low compared with our nCT regimen, and we think the low dose of paclitaxel cannot bring a high rate of pCR and, ultimately, cannot bring a survival benefit. The JCOG1109 study is also a multicentre RCT to compare the treatment of preoperative chemotherapy with cisplatin and 5-FU and a 3-drug combined preoperative chemotherapy regimen with the addition of docetaxel and preoperative chemoradiotherapy for patients with locally advanced ESCC [25]. A total of 501 patients will be accrued from 41 Japanese institutions within 6.25 years. Different from the JCOG1109 trial, we used paclitaxel and cisplatin as the nCT regimen mainly because of the acceptable toxicity and high pCR rate. In our previous retrospective study, the overall pCR rate was 20.5% for the nCT regimen with paclitaxel plus platinum in patients with locally advanced ESCC [26].

## Conclusion

Overall, there is currently no large-scale study that has aimed at a direct comparison between nCRT with the CROSS regimen and nCT in patients with ESCC, and there is no strong evidence about the feasibility and safety of the CROSS regimen for patients with oesophageal cancer in China. We believe it is very important to compare the CROSS regimen with nCT in China, where ESCC patients account for more than 90% of all oesophageal cancer patients. The results of HCHTOG1903 will provide enough information about the controversy regarding whether nCRT is superior to nCT alone in patients with ESCC.

## Supplementary information

**Additional file 1.** Definition of complications.

## Data Availability

Not applicable.

## Abbreviations

AE: Adverse events
ALT: Alanine transaminase
ANC: Absolute neutrophil count
AST: Aspartate transaminase
AUC: Area under the curve
CBP: Carboplatin
CRE: Clinical response evaluation
CT: Computed tomography
CTV: Clinical target volume
DDP: Cisplatin
DFS: Disease-free survival
ECG: Electrocardiogram
EORTC: European Organization for Research and Treatment of Cancer
ESCC: Oesophageal squamous cell carcinoma
ITT: Intention-to-treat
JCOG: Japanese Clinical Oncology Group
MDT: Multidisciplinary team
MIE: Minimally invasive oesophagectomy
nCRT: Neoadjuvant chemoradiotherapy
nCT: Neoadjuvant chemotherapy
NRS: Nutritional risk screening
OS: Overall survival
pCR: Pathological complete response rate
PET: Positron emission tomography
PTX: Paclitaxel
QOL: Quality of life
RCT: Randomized controlled trial
ULN: Upper limit of normal
WBC: White blood cell
