# Supplementary material for: Neoadjuvant chemotherapy versus neoadjuvant chemoradiotherapy for locally advanced oesophageal squamous cell carcinoma: a single-Centre, open-label, randomized, controlled, clinical trial (HCHTOG1903)
Source: BMC Cancer. 2020 Apr 15;20:303. doi: 10.1186/s12885-020-06824-2 (PMC7158003; doi:10.1186/s12885-020-06824-2)
Supplement: Supplementary file 1 — Additional file 1. Definition of complications. [file 12885_2020_6824_MOESM1_ESM.docx]

Additional file 1: Definition of complications

| **Complication** | **Criteria** |
| --- | --- |
| ***Cardiac Complications*** |  |
| Cardiac infarction | Confirmed by electrocardiography or echocardiography and cardiac enzyme monitoring |
| Heart failure | Confirmed by echocardiography or necessitating pressure agents |
| Arrhythmia | ECG confirmed and necessitating medication |
| Pericarditis | Diagnosed by pericardiocentesis and requiring treatment |
| Cardiac tamponade | Diagnosed by echocardiography and requiring treatment |
| ***Respiratory Complications*** |  |
| Pneumonia | X-ray or CT confirmed and necessitating antibiotic treatment |
| Airway necrosis or fistulae | Confirmed by endoscopy |
| Respiratory failure | Need for mechanical ventilation for greater than 24 h in a patient who requires reintubation after surgery OR need for mechanical ventilation for greater than 72 h in a patient who is not extubated on the day of surgery [1] |
| ARDS | Berlin definition [2] |
| Atelectasis | X-ray or CT confirmed and requiring bronchoscopy |
| Air leak | Chest tube maintenance for air leak for > 7 days postoperatively |
| Pulmonary embolus | Confirmed by angio-CT scan |
| Pleural effusions | X-ray or CT confirmed and requiring treatment |
| Chest infection | Supported by positive bacterial culture |
| Pneumothorax | X-ray or CT confirmed and requiring treatment |
| Mediastinitis | Supported by positive bacterial culture |
| ***Gastrointestinal complications*** |  |
| Anastomotic leak | Extravasation of water-soluble contrast during a swallow study, visualization of either anastomotic dehiscence or fistulae during endoscopy or visible loss of saliva or methylene blue through the cervical wound |
| Nonanastomotic leak | Radiologically or endoscopically identified |
| Conduit necrosis | Endoscopically or intraoperatively identified |
| Intra-abdominal abscess | Supported by positive bacterial culture |
| Peritoneal effusion | Doppler ultrasound or CT confirmed and requiring treatment |
| Gastrointestinal bleeding | Requiring intervention or transfusion |
| Liver failure | Need for FFP to correct INR in patient with serum bilirubin > 12 mg/dL OR INR > 2.5 in a patient with serum bilirubin > 12 mg/dL^1^ |
| Intestinal obstruction | Clinical symptoms, radiological confirmation and needing treatment |
| Delayed gastric emptying | Clinical symptoms, radiological confirmation and needing treatment |
| Diaphragmatic hernia | Diagnosed by barium swallow or CT scan and then confirmed intraoperatively |
| **Other complication** |  |
| Transient ischemic attack | Defined according to American Heart Association/American Stroke Association expert consensus |
| Cerebral infarction | CT or MRI confirmed |
| Cerebral hemorrhage | CT or MRI confirmed |
| Urinary tract infection | Presence of microorganisms in the urine accompanied by one or more of the following: dysuria, urgency, loin pain, tenderness, pyrexia or pyuria |
| Urinary retention | Requiring reinsertion of urinary catheter |
| Acute renal insufficiency | Doubling of baseline creatinine |
| Renal failure | Need for dialysis in a patient not on dialysis preoperatively |
| Wound infection | Requiring opening of wound or antibiotics |
| Bacteremia | Supported by at least one blood culture positive for pathogenic organisms |
| Sepsis | Temperature > 38°C or < 36°C, heart rate > 90 beats/min, WBC count > 12000 cells/mL or < 4000 cells/mL, and bacteremia |
| Postoperative hemorrhage | Postoperative bleeding > 2000 mL or need for reoperation |
| Recurrent laryngeal nerve paralysis | Identified by endoscopic examination |
| Chylothorax | Increase in chest tube output with enteral alimentation, change in the nature of the output to a milky appearance, and confirmation by a physical-chemical analysis of the fluid |
| Deep venous thrombosis | Clinical symptoms and vascular color echo-Doppler diagnosis |
| Fat necrosis | Clinical symptoms and exception of infection |
| Subcutaneous effusion | Clinical symptoms and requiring treatment |

Abbreviations: ECG = electrocardiogram; CT = computed tomography; ARDS = acute respiratory distress syndrome; FFP = fresh frozen plasma; INR = international normalized ratio; MRI = magnetic resonance imaging.

**REFERENCES**

1. Strasberg SM, Linehan DC, Hawkins WG. The accordion severity grading system of surgical complications. Ann Surg. 2009;250(2):177-86.

2. Ranieri VM, Rubenfeld GD, Thompson BT, Ferguson ND, Caldwell E, Fan E, et al. Acute respiratory distress syndrome: the Berlin Definition. JAMA. 2012;307(23):2526-33.
